# Taylorism on steroids or enabling autonomy? A systematic review of algorithmic management

**DOI:** 10.1007/s11301-023-00345-5

**Published:** 2023-04-05

**Authors:** Niilo Noponen, Polina Feshchenko, Tommi Auvinen, Vilma Luoma-aho, Pekka Abrahamsson

**Affiliations:** 1 School of Business & Economics, University of Jyväskylä, P.O. Box 35, Jyväskylä, 40014 Finland; 2 Faculty of Information Technology and Communication Sciences, University of Tampere, Tampere, Finland

**Keywords:** Algorithmic management, Algorithms, Digital Taylorism, Paradox of autonomy, Information asymmetry, Digital labor, Systematic literature review

## Abstract

The use of algorithmic management systems is rapidly changing organizational models and practices, as millions of workers in multiple sectors worldwide are managed by computer software. Despite receiving increasing academic interest, little summarizing literature exist on the ways algorithmic systems are used in management. This article aims to fill this gap by systematically reviewing and qualitatively analyzing 172 articles on the topic. Our research contributes to the existent algorithmic management literature in three ways. First, we provide a descriptive overview of algorithmic management as a field of research. Second, we identify and synthesize the discussion on the key concepts of the topic, namely how algorithmic management: (1) simultaneously restrains and enables workers’ autonomy—yet income dependency and other factors force inflexible work practices; (2) creates a complex, digital version of Taylorism; and (3) creates new and changes existing organizational power structures. Third, as our main theoretical contribution, we create the framework of Algorithmic Management Grid that demonstrates the ways in which organizations use algorithmic systems in management. The Algorithmic Management Grid illustrates how, in both novel and traditional organizational models, algorithmic management may be used with emphasis either on controlling or enabling workers. Based on the reviewed literature, we claim that so far, companies have mostly utilized algorithmic systems in a controlling manner, neglecting the technology’s enabling potential in organizing.

## Introduction

Algorithmic systems are used in managing millions of workers in organizations around the world. According to an estimate by Kässi, Lehdonvirta, and Stephany (2021), the number of people who have obtained work through online freelancing platforms is 19 million. The COVID-19 pandemic seems to have further increased the number of people impacted by algorithmic management techniques, both in platform work, such as food delivery and ride-hailing, as well as in more traditional forms of employment. According to a Guardian report (Marks 2021), the number of companies choosing to use algorithmic means of monitoring remote office workers is also expanding.

Algorithmic management was first defined by Lee et al. (2015) as “software algorithms that assume managerial functions and surrounding institutional devices that support algorithms in practice.” In the evolution of organizational theory, algorithmic management seems to represent something new, yet some of its aspects are deeply rooted in the management tradition. For the first time, organizations are able to oversee workers without the need for human intervention. Yet, this form of control seems to have been modeled after factories in the 19th century. Labeled scientific management 2.0 (Schildt 2017), this digital version of Taylorism also seems to consist of a high degree of standardization, de-composition, digital surveillance, and measurement of labor (Altenried 2020).

Similarly, Huws (2016) suggests that across all sectors of the economy, from low-skilled to high-skilled or from public to private, a new paradigm of work organization called ‘logged labor’ has been brewing. In this model, work is cut up into standard, quantifiable components, subjected to continuous surveillance and monitoring, and requires the worker to be connected to an online platform to obtain work. Although these are most evident in short-term hires or ‘gig economy’ companies, such as Uber and Amazon Mechanical Turk, similar trends are increasingly observable in more traditional organizations as well. According to Fleming (2017), this “Uberization” is a consequence of neoclassical economic ideas, such as human capital theory, which place responsibility and risk on the shoulders of individualized workers. Paradoxically, autonomous, self-managed workers face more management and control than before (Fleming 2017, p. 702), often enabled by algorithmic systems.

According to Faraj, Pachidi, and Sayegh (2018), due to algorithms’ ability to reshape work and organizing, “the extent to which the algorithm is authorized to make decisions, the need to incorporate morality in the technology, and their digital iron-cage potential” need more scholarly attention. With ever-present tracking and monitoring, the logic of surveillance capitalism is stretched not only to work—but to seemingly all aspects of human behavior (Zuboff 2019). In this context, algorithmic management has emerged as a tool that facilitates completely new forms of managing and organizing.

Therefore, to better understand this phenomenon that appears to have the potential to shake up organizational foundations, a systematic literature review is in order. A systematic literature review has key advantages as a research method. A literature review can map and assess the existing intellectual territory (Tranfield et al. 2003, p. 208). Literature reviews also facilitate theory development and create a foundation for advancing knowledge (Webster and Watson 2002). According to Snyder (2019), as interdisciplinary knowledge production accelerates, literature review as a research method becomes more relevant than ever. Algorithmic management is not an exception. Although defined as recently as 2015, the topic has received increasing interest among diverse fields of research from sociology to computer science. However, due to its interdisciplinary nature, the research on the topic is scattered among various journals in different fields of inquiry. As such, the research area of algorithmic management is at a stage where a systematic literature review is needed to combine, analyze, and synthesize the numerous insights provided by previous studies on the topic.

In this study, our aim is to examine how algorithmic systems are used in management. To fulfill our research aim, we have executed an analysis consisting of three parts: (1) to map the terrain, we produce a brief descriptive overview of previous literature, (2) we identify and analyze the key concepts among the scholarly discussion on algorithmic management, and (3) we synthesize the findings of the previous stages to construct a framework of the ways that algorithmic systems are used in management, the Algorithmic Management Grid.

The paper is structured as follows. After introducing algorithmic management through the evolution of its definitions, we uncover the methods and steps taken in conducting a systematic literature review. In the section that follows, we first describe the literature in general terms, before moving to an in-depth analysis of the key concepts, specifically how algorithmic management simultaneously restrains and enables autonomy, creates a digital version of Taylorism, and creates new and changes existing power structures. The scholarly debate on each concept is examined in depth. Next, with the help of our framework of the Algorithmic Management Grid, we claim that there are various ways of using algorithmic management, out of which most of the case organizations in the literature have chosen a more controlling than enabling model. Finally, before our concluding remarks, we discuss the wider implications of the findings of our literature review and of the Algorithmic Management Grid, while proposing possible avenues for future research.

## Background literature

### Development of the definition of algorithmic management and our approach to the topic

Scholars have used slightly different definitions of algorithmic management, based on how specific each definition is. The first and perhaps most used definition represents a broader version, which Lee et al. (2015, p. 1603) describe as “software algorithms that assume managerial functions and surrounding institutional devices that support algorithms in practice algorithmic management.” Kaine and Josserand’s (2019) further generalized their definition as “management of labor by machine.” The benefit of a broad definition is that it skips the often-endless debate common for example in determining what is and is not artificial intelligence – as according to this definition, if technology is used to carry out any managerial task, it may be considered algorithmic management. From a management perspective this matters more than defining different systems based on their technological sophistication, for example.

Möhlmann and Zalmanson (2017, p. 4) developed a more specific version from an information systems science perspective, defining algorithmic management “as oversight, governance and control practices conducted by software algorithms over many remote workers.” They further identified five characteristics of algorithmic management: (1) constant tracking of workers’ behavior, (2) constant performance evaluation, (3) automatic implementation of decisions, (4) workers’ interaction with a “system,” and (5) (low) transparency.

Along similar lines, Jarrahi and Sutherland (2019, p. 579) referred to algorithms’ capacity to manage large groups of workers, as they referred to algorithmic management as having “emerged in digital labor platforms as a method of organizing and coordinating extremely large groups of workers and clients in an automated way.” A key aspect of this definition is digital labor platforms, an aspect that Jarrahi et al. (2020) further distinguish by coining the term platformic management to emphasize the dynamic interaction between people and technology. In their definition, Duggan et al. (2020) also point to the cyclical nature of algorithmic management: algorithms that control workers are developed by data from the same workers.

In a more recent article, authors who had previously used alternate definitions – Lee and Jarrahi, Sutherland, and Kinder, alongside Newlands (2021) – came together for a sociotechnical definition of algorithmic management. The authors referred to Jarrahi and Sutherland’s (2019) article, stating that “algorithmic management should rather be understood as a sociotechnical process emerging from the continuous interaction of organizational members and the algorithms that mediate their work.” This definition emphasizes that algorithms are not autonomous technological entities, as algorithmic management is a sociotechnical concept shaped by both social and technological forces. The authors stress that workers’ and managers’ organizational choices and competencies shape algorithms that manage work, which in turn shifts organizational roles and power relationships, affecting workers and managers.

Similar to Jarrahi et al. (2021), we examine the topic through the framing of algorithms as a social construct. In other words, we do not view algorithms as mere technology, but rather as diverse sociotechnical systems created in the complex interaction between the algorithm and different organizational actors. The manifestation and consequences of a particular algorithmic system are shaped by the people developing, commissioning, using, or being managed by the system, as well as its place in the wider organizational environment (Jarrahi et al. 2021).

### Previous review articles on algorithms in management and related topics

Algorithmic management represents a topic of inquiry that is relatively clearly defined yet has many extensions to related topics. Previous review articles have focused on the overlapping, yet separate topics, such as algorithms in human resource management (Cheng and Hackett 2021; Meijerink and Bondarouk 2021), the interplay of artificial intelligence and strategic management (Keding 2021), dynamics and digitalization in performance management systems (Sahlin and Angelis 2019) and human trust in artificial intelligence in an organizational context (Glikson and Woolley 2020). Numerous review articles have also been published on the relatively novel area of gig/sharing economy, in which the role of algorithmic management is perhaps most salient. Review articles have been published on gig economy in general (Cheng 2016; Agarwal and Steinmetz 2019), in computing (Dillahunt et al. 2017), its definitions (Görög 2018; Weili and Khan 2020), its organization and experience of work (Kaine and Josserand 2019) and its business models, operational insights, and environment-based utilities (Zhu and Liu 2020).

However, despite the timeliness of the topic, reviews of algorithmic management itself have remained scarcer. Recently, Kellogg, Valentine, and Christin (2020) presented an extensive review on the topic of algorithmic control, which can be understood as one of the five functions of algorithmic management following Fayol’s (1917) classification. Kellogg et al. (2020) contribute considerable insights to the study of algorithms by focusing on how algorithms are used as a form of rational control and by identifying the mechanisms of algorithmic control: restricting and recommending, recording, and rating, as well as replacing and rewarding. The authors established key insights from the literature: (1) labor process theory highlights potential problems with mainly positive views of algorithms at work; (2) algorithmic control is distinct from old ways of (technical and bureaucratic) rational control; (3) the use of algorithms is creating new occupations; and (4) workers resist algorithmic control individually and collectively using tactics that scholars label as algoactivism. Kellogg et al. (2020, pp. 5–7) also cover some of the technological affordances of algorithmic systems.

Furthermore, through the lens of self-determination theory, Gagné et al. (2022) conducted a review of how algorithmic management influences worker motivation. The scholars found a mostly negative effects of the use of algorithmic management on worker motivation and need satisfaction, although moderating effects do exist through management features and practices (Gagné et al. 2022).

In turn, Parent-Rocheleau and Parker (2022) reviewed algorithmic management literature to examine how algorithmic systems influence the design of jobs. Parent-Rocheleau and Parker (2022) developed a model that synthesizes six key managerial functions in which algorithms have been used: (1) monitoring, (2), goal setting, (3) performance management, (4) scheduling, (5) compensation, and (6) job termination.

In their review article, Heinrich, Minh Anh and Vysochyna (2022) developed a taxonomy to classify five areas of algorithmic management: (1) mechanisms, (2) effects, (3) concerns and (4) design of algorithmic management, as well as (5) second party’s (workers’) response to algorithmic management. The authors provide valuable contributions to the topic, yet as their approach is from an information systems science perspective, the understanding of management as a field of research is at times lacking in the article. This is evident, for example, in the explicit treating of the terms algorithmic management and algorithmic control as interchangeable synonyms (Heinrich et al. 2022, p. 2), instead of viewing the former as the hypernym of the latter. In addition, in a recent conference paper, Cameron et al. (2023), evaluated algorithmic management’s implications for information systems research, as well as possible future directions.

Despite the aforementioned reviews of algorithmic systems in different contexts, previously there has been no systematic literature review article that takes a concept-centric approach (Fisch and Block 2018) towards examining algorithmic management from an organization studies perspective. We believe there exists a need to identify, analyze and synthesize the key concepts among the scholarly discussion on algorithmic management in various research fields – sociology, management, information systems science, and computer science, among others – in order to create new theoretical insights, as well as to provide guidance for future research.

## Methods: systematic literature review

This study adopts a qualitative systematic literature review method. We conducted the review following the general guidelines stipulated by Snyder (2019) and the more specific guidelines for each research process by Tranfield, Denyer, and Smart (2003).

Furthermore, we analyzed the articles using the concept-centric approach suggested by Webster and Watson (2002) and Fisch and Block (2018). We identified and evaluated the underlying concepts of algorithmic management, which, in turn, guided the analysis. According to Fisch and Block (2018), focusing on concepts instead of individual studies helps to identify the research debates that authors aim to contribute to and helps to ensure a better structure throughout the article. Another benefit of the concept-centric approach is that it helps in finding a right balance between breadth and depth, as in a good systematic literature review all relevant studies are included, but only the most important studies are described in detail and in a structured manner (Fisch and Block 2018). Following the concept-centric approach can also lead to a new conceptual framework, which makes writing a systematic literature review similar to writing a conceptual theory paper (Fisch and Block 2018). As such, the approach is suitable to achieving the research aim of this article.

Tranfield et al. (2003) divide the process of conducting a review article into three major stages: (1) planning review, (2) conducting review, and (3) reporting and dissemination. Before the beginning of the review process, a scoping study was completed, and a clear set of search terms was defined to provide a cross-disciplinary perspective on the topic. As such, relevant articles were searched with the following keywords: “algorithmic management”, “algorithmic leadership”, “algorithmic labor”, and “algorithmic labour.” The first keyword is chosen because the focus of our study is the relatively defined topic of algorithmic management. The three other keywords were chosen to include discussion on related areas. Five academic databases were chosen for the review to ensure the inclusion of contributions from various fields of science—Scopus, Web of Science, EbscoHost, Proquest, and JSTOR.

We included only peer-reviewed academic literature available in English. In addition to journal articles, peer-reviewed conference papers were included, as they are a standard form of publishing in the fields of computer science and information systems science, both of which have made key contributions to the algorithmic management literature. In order to be considered relevant, an article has to make a contribution to the scholarly discussion on algorithmic management. This contribution can naturally take many forms, from novel theoretical frameworks to providing empirical insights of algorithmic management in different contexts. We did not use a journal ranking threshold, as we wanted to include any contribution to the discussion on algorithmic management made in articles published by less famous journals as well.

Our search terms provided the initial crop of 251 articles. The first stage of reading the articles focused on the abstracts, and the goal was to identify key information for each article (year, citation count, case, country, conceptual/empirical, method, journal, area of research, research question, contribution), which was systematically recorded in a comprehensive review document. We excluded 44 articles in medical science and psychology that refer to a different meaning of algorithmic management, e.g., “algorithmic management of pediatric acute mastoiditis”.

The second stage of reading was based on an extensive examination of the articles in full while listing the key concepts arising from the literature. Further, 35 articles that did not contribute to the discussion on algorithmic management was excluded in this stage. These articles include, for example, introductory articles of a special issue that set the scene for following articles, rather than offering novel insights on algorithmic management. As a result of this process, the total number of articles included in the review is 172. The research process is outlined in Table 1.

**Table 1:** Research process

1. Initial scoping study
2. Identifying keywords • “algorithmic management” OR “algorithmic leadership” OR “algorithmic labor” OR “algorithmic labour” • Databases: Scopus, Web of Science, EbscoHost, Proquest, and JSTOR
3. Setting the inclusion and exclusion criteria • Only peer-reviewed journal articles and conference papers (no journal ranking used) • English language • Contributes to the discussion on algorithmic management
4. First round of reading the articles – abstract • 251 articles in total • Including only relevant articles – excluding 44 articles on medical science with a different meaning of algorithmic management • Establishing key information • Year, citation count, case, country, conceptual/empirical, method, journal, area of research, research question, contribution
5. Second round of reading the articles – full text • Identifying and compiling a list of key concepts by thoroughly examining all articles • Excluding 35 articles that do not contribute to the discussion on algorithmic management • 172 articles in total (March 2023)
6. Analysis • Forming tables and graphs to support analysis • Separating concepts to the first and second orders

ANALYZING THE LITERATURE ON ALGORITHMIC MANAGEMENT

### Descriptive overview

**Fig. 1:**
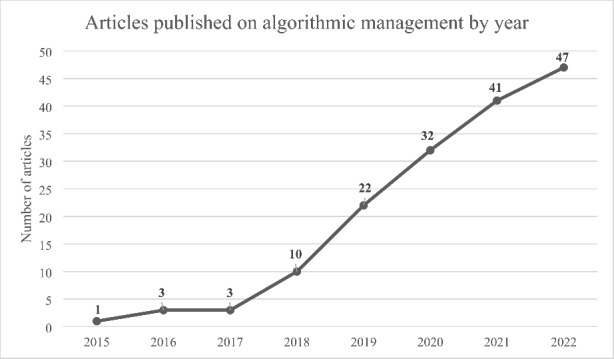
The number of articles published annually on algorithmic management

Algorithmic management is a topic that has received increasing academic attention in recent years. Since Lee et al. (2015) defined the concept in an organizational studies context in 2015, each year has seen at least as many publications as its predecessor. The number of peer-reviewed articles published on algorithmic management each year is visualized in Fig. 1.

The topic has received interdisciplinary attention, mainly from sociology, management, information systems science, and computer science. Overall, the literature on algorithmic management can be described as fairly critical of the way case organizations have utilized algorithms to manage workers. Existing studies are characterized by concern for the people working under the novel method of control and organizing. This perhaps explains why the level of disagreement on the topic has been surprisingly low, at least in the tradition of organizational and social science. Although discussion on some concepts regarding algorithmic management is more divided than on others, the literature has so far been more complementary than refuting in nature.

Schor et al. (2020, pp. 835–837), however, make the distinction between two major camps: precarity scholars and algorithmic control scholars. Precarity scholars view platform labor as a part of a “great risk shift,” a return to 19th century market-based processes of organizing labor, as opposed to the more stable and secure employment of the post-war period. Characteristic of this trend in platform companies is that the workers, classified as independent contractors, assume most risk of downturns; however, if the company does well, only the shareholders benefit financially. By contrast, according to Schor et al. (2020), algorithmic control scholars emphasize discontinuity with previous systems, viewing algorithmic technologies as distinguishing platforms from traditional companies. It is not clear, however, how pronounced this distinction between precarity and algorithmic control scholars is. We would argue that what is in question is more of a different point of emphasis rather than two incompatible schools of thought.

The focus of research has not been as much on the technology itself, but rather on workers’ perceptions of the way algorithms have been used in managing them. The starting point for many researchers seems to have been “Yes, algorithmic management does have novel affordances – but at what human cost?” In other words, the literature has had an eye on the trade-offs of algorithmic management, especially from the workers’ point of view. Hence, many case studies have focused on workers’ experiences of algorithmic management, as well as on related ethical issues.

Across multiple studies, researchers have highlighted how algorithmic management may not afford the flexible autonomous work that companies advertise as well as how algorithms may know everything about the worker, yet the worker knows nothing of the algorithm. This one-way transparency seems to be key in creating information and power asymmetries between workers and companies. Another area of interest has been studying the ways companies use algorithms to control the workforce (which is reviewed and summarized by Kellogg et al. 2020), as well as the ways workers defy, resist, and work around the algorithms.

During the early years of algorithmic management research (roughly between 2015 and 2020, most empirical research was based on the platform economy, with ride-hail, food delivery, and freelancing companies commanding the most attention. This is certainly no surprise, as algorithmic management seems most prominent in the relatively novel and constantly growing sector. While Uber is the case organization on which algorithmic management scholars have focused the most, there are more than just a few players in the algorithmic field. Indeed, case studies on the topic vary from beauty workers in India (Anwar et al. 2021) to food delivery workers in Europe (e.g., Galière 2020; Kusk and Bossen 2022).

Conversely, the use of algorithmic management systems in more traditional work contexts had received less consideration before 2021, as emphasized by Jarrahi et al. (2021). However, since then, scholars have widened their scope beyond platform companies, including more diverse contexts in the empirical record. For example, studies have focused on how digital control methods impact credit agents’ labour process in a lending company (Terry et al. 2021), how algorithms can be used to create diverse teams within an organization(Gómez-Zará et al. 2022) and how co-designed algorithmic systems can be used in responsible governing of a refugee camp (Dekker et al. 2022). It should also be noted that it is entirely possible that scholars have not used the term algorithmic management when studying digital management technologies in traditional office settings.

To summarize, algorithmic management has been established through numerous studies as a central factor in the way work is organized now and in the future. In less than a decade, algorithmic management has evolved into a multi-disciplinary research area of considerable, yet not full maturity, encompassing an expanding variety of empirical contexts.

### Key concepts in algorithmic management literature

Following Fisch and Block (2018), we structured our analysis based on concepts, instead of individual articles. We have compiled the concepts we identified as most pivotal among the discussion on algorithmic management in Table 2. In this chapter, we analyze each concept in detail.

**Table 2:** Key concepts among algorithmic management literature

	Concept	Description	Key sources
1	**Paradox of autonomy & income dependency**	Algorithmic management paradoxically restrains and enables autonomy simultaneously • Autonomy afforded o Choosing when and where to work o Gaming & working around the algorithm • Autonomy restricted o Close control & monitoring • More income dependency of platform labor → less autonomy • Forced to specific time, place, and methods of work to maximize income (digital iron cage)	Lee et al. 2015, Möhlmann and Zalmanson 2017, Ma and Hanrahan 2019, Wood 2019, Jarrahi 2019, Schor et al. 2020, Bokányi & Hannák 2020, Anwar et al. 2020, Jarrahi et al. 2020, Reid-Musson et al. 2020, Rani & Furrer 2020, Galière 2020, Meijerink and Bondarouk 2021, Ruiner and Klumpp 2022, Perez et al. 2022
2	**Digital Taylorism**	Algorithmic management creates a deeper version of Taylorism • Familiar characteristics o Piece-meal wage o Standardization o Deskilling o Exploitation • New Characteristics: o “Assembly line” to new professions o Digital scaffolds instead of physical control o Global heterogeneous workforce instead of digital mass workers o Rational control supported by normative control & entrepreneurial rhetoric	Rosenblat & Stark 2015, Schildt 2017, Altenried 2019, Wood et al. 2019, Jarrahi 2019, Mengay 2020, Ovetz 2020, Jarrahi et al. 2020, Veen et al. 2020, Altenried 2020, Reid-Musson et al. 2020, Stark and Pais 2020, Altenried 2021, Pignot 2021, Braesemann et al. 2022, Schaupp 2022
3	**Power structures & Information asymmetry**	Algorithmic management creates new and changes existing power structures • Power shifts from (middle & operative) managers to experts in programming, analytics, & artificial intelligence • Information asymmetry: algorithms know everything about workers; workers know little of algorithms	Rosenblat and Stark 2016, Schildt 2017, Newlands et al. 2018, Chan and Humphreys 2018, Gandini 2019, Jarrahi 2019, Wood et al. 2019, Kinder et al. 2019, Jarrahi et al. 2020, Altenried 2020, Stark and Pais 2020, Bucher et al. 2021, Aloisi and De Stefano 2022, Barati and Ansari 2022

#### The paradox of autonomy and how income dependency impacts the experience of working under algorithms

It has been known for some time that information technology has the capacity to overturn hierarchical social orders and foster a more democratic organizational culture by creating more equal access to information, as Jarrahi (2019, p. 185) points out, referring to Zuboff (1988). Nevertheless, Jarrahi states that this informing power may also be abandoned, leaving opaque algorithms to reproduce existing organizational structures. Whether algorithmic management is used in a controlling, or an enabling manner is crucial to how it is experienced.

The nature of algorithmic management practices in contemporary platform companies appear more controlling than enabling, as many jobs largely consist of validating the performed micro-tasks on the platform. As a worker interviewed by Galière (2020, p. 364) stated, “I feel like I spend all my time confirming everything I do. I confirm that I’ve put the pedal to the left, I confirm that I’ve put the pedal to the right…”.

Whether algorithmic management affords freedom, flexibility, and autonomy has been one of the central questions on the topic. To recruit workers, most platform companies urge to “be your own boss, decide when and where you want to work.” Although the statement may have a hint of truth in it, the empirical evidence of it appears questionable in many cases. It seems that there are simultaneously certain factors that enable autonomous work and others that diminish it. For example, in their study of online freelancing platforms, Wood et al. (2019) found that algorithmic management techniques “tend to offer workers high levels of flexibility, autonomy, task variety and complexity,” but as a trade-off, “these mechanisms of control can also result in low pay, social isolation, working unsocial and irregular hours, overwork, sleep deprivation and exhaustion.” However, Bucher, Schou, and Waldkirch (2021, p. 17) question even this level of autonomy. Although they acknowledge the platform workers’ freedom to choose their own schedule and their clients, Bucher et al. (2021) stress that the platform severely constrains the client-worker relationship, since workers must please both the client and the algorithm to make sure they are able to continue participating on the platform.

Möhlmann and Zalmanson (2017) call the tension between workers’ sense of autonomy and the algorithmic systems’ need for control a paradox of autonomy. Similarly, Meijerink and Bondarouk (2021) build on previous studies to emphasize how human resource management algorithms simultaneously restrain and enable worker autonomy and value. This perception of autonomy appears to vary between companies as well as among workers in the same company.

Perhaps the most crucial determinant of perceived autonomy in algorithmically managed platform work is the worker’s income dependency on the job. Muralidhar, Bossen and O’Neill’s (2022) case study of the Indian ride-hailing platform Ola encapsulates the phenomenon. The authors describe how the platform and its design (1) heighten Ola drivers’ precarity while providing little benefits and (2) shift more customers from street-hailing to app-based hailing, which intensifies the drivers’ dependence of the platforms (Muralidhar et al. 2022). Similarly, Ma and Hanrahan (2019) found that dependency on platform income heavily influenced Uber drivers’ attitude toward the platform and difficulties faced on the job. Schor et al. (2020) emphasized this heterogeneity of the workforce in various platform companies as well as the disparate experiences of part-time and full-time gig workers. Schor et al. (2020) also found that, to have satisfying work experiences, alternative sources of income and security were almost a precondition, as supplemental earners were more satisfied and earned more. Furthermore, Wood et al. (2019) assess that on online freelancing platforms, most workers have two options: (1) low flexibility, high intensity, and high job security; or (2) high flexibility, low intensity, and low job security.

According to Rani and Furrer’s study (2021), on five online freelancing companies—Amazon Mechanical Turk, CrowdFlower (Figure Eight), Clickworker, Microworkers, and Prolific—the average earnings were $2.69 per hour (and $2.03 when unpaid work was accounted for). The authors conclude that the design features of these platforms do not allow workers any control or autonomy over their work process or working time in this context of online freelancing in developing countries. Furthermore, the global workforce established by these micro task platforms seems to create “a race to the bottom,” where the one asking for the smallest compensation will receive the job. Rani and Furrer (2021, pp. 20–21) point to similarities between earlier forms of business process outsourcing that created routine, unspecialized jobs in indecent working conditions.

While algorithmic systems’ impact on worker autonomy is most pronounced in platform work, similar trends have been observed in more traditional fields as well. For example, Ruiner and Klumpp (2022) report how the previously autonomous job of truck drivers has seen increasing levels of control and transparency through the introduction of algorithmic systems, such as digital cargo scanners and location and downtime tracking tools. Similarly, Perez, Conway, and Roques (2022) describe how bank employees felt their autonomy decreased after the bank started using machine learning systems as a part of their work tasks.

For full-time platform workers, the experience of control appears more intense: to make a living, they are often forced to specific practices over which they have little control. These practices include long workdays, finding ways to game, circumvent, or manipulate the algorithm, and tricks specific to each gig job, such that algorithmic management may create a digital version of Taylorism. Based on the literature, flexible and autonomous platform work appears elusive, especially if the worker is entirely dependent on platform income.

#### Digital Taylorism – Are algorithms taking management back a hundred years?

Scholars argue that with algorithmic management practices, new professions are introduced with typical aspects of Taylorism, from piecemeal wage to standardization, deskilling, decomposition, commodification, and exploitation of work (Schildt 2017; Altenried 2019; Jarrahi 2019; Mengay 2020). In contemporary organizational theory, this phenomenon, called digital Taylorism, appears to represent a new direction with some familiar characteristics.

Stark and Pais (2020) point out how in the early twentieth century Taylorism legitimated the growth of a new managerial class. In addition to Taylorism’s traditional image of the assembly line, computer integrated manufacturing (CIM) and its vision of automating control functions during the 1970 and 1980 s, as well as the lean production methods and their digital performance measurement systems that became widespread by the 1990s can be seen as early versions of algorithmic management (Schaupp 2022). Such control-oriented algorithmic management practices appear to reinforce the mechanistic idea of man inherent in scientific management (see e.g., Grint 2011), in which a worker is a standardized, replaceable cog in the operating system. Illustrating the phenomenon, Altenried (2019) describes how UPS delivery drivers face increased measurement, intensification, and surveillance of labor with a 74-page guidebook and 200 sensors per truck guiding their work.

One could claim that the original Taylorist mode of organizing work is already algorithmic in nature (following a specific list of orders), but the performance is still overseen by a human manager. In digital Taylorism, humans perform algorithmic work while being supervised by software algorithms, who possess no consideration for the workers humanity that a human supervisor may have. Consequently, Altenried (2019) argues that instead of a mere rebirth of Taylorism, digital technologies extend and radicalize the logics that have been at work for centuries.

Not limited to factories and warehouses, the digital version of Taylorism appears to be entering new fields in new ways. Ovetz (2020, p. 2) notes how standardization, deskilling, and commodification have entered academic work with the introduction of “education technologies designed to rationalize academic labor by transforming the assessment of comprehension of content knowledge to measurement of proficiency in task completion”. Giermindl et al. (2021, pp. 16–17) point out how transferring the efficiency-driven logic of analytics to human resource management carries numerous risks, from impairing transparency and accountability to marginalizing human reasoning and eroding managerial competence.

Remote freelancing and crowdwork platforms also recreate new forms of Taylorism. According to Altenried (2020), instead of producing a “digital mass worker,” these labour platforms create a heterogeneous global workforce. Braesemann et al. (2022) observe similarities in the spatial division of work between this trend and the offshoring of business process that started in the 1980s; in this case, how increasingly standardized and modularized knowledge work tasks enable the global division of knowledge work between the global north and global south. According to Braesemann et al. (2022) digitally enabled remote work is likely to reinforce the prevalent global dynamics rather than distributing knowledge work more evenly.

Given that the clients of remote freelance workers may live on different continents, workers may experience relative freedom and autonomy in how to work, as long as the end product is satisfactory (Wood et al. 2019, pp. 64–65). Still, intense, irregular work schedules seem common, and workers may be expected to work in the middle of the night. According to Wood et al. (2019), in this remote freelance work context, contrary to traditional forms of Taylorism, control is enforced at the end of the labor process rather than during it. Indeed, modern forms of Taylorism do not necessarily depend on physical control but on digital scaffolds of monitoring and control (Rosenblat and Stark 2016, p. 3772). Altenried (2020) argues that such measures build a figurative factory of digital labor, which seems to mirror the concept of “digital iron cage,” as phrased by Faraj, Pachidi, and Sayegh (2018).

As a part of the digital scaffold, researchers have noted the importance of the rating system, which outsources performance evaluation to the customer and may even automatically fire workers if their performance drops under a certain level (Lee et al. 2015; Rosenblat and Stark 2016). Moreover, drawing on labor process theory in his analysis of platform companies, Gandini (2019) argues that labor power “comes to be transformed into a commodity in a context where the encounter between supply and demand of work is mediated by a digital platform, and where feedback, ranking, and rating systems serve purposes of managerialisation and monitoring of workers”.

According to Galière (2020), in addition to rational control, normative control also plays a part. In her study, Galière (2020) uses the case of Deliveroo in France to show how self-entrepreneurial discourse may be used to foster acceptance of algorithmic management. Similarly, Pignot (2021) stresses how crucial ideology is to organizational control and how ideologically seasoned algorithms may go deeper than traditional control mechanisms.

With such an array of control methods, standardized algorithmic management practices seem to eradicate or circumvent the need for trust. Whether ordering a food delivery, a ride across town, or data labeling services, the clients (and employers) know that they will receive a standardized service. For the person offering these services, however, this often means less creativity or even the need to make decisions, and more following detailed instructions and ticking boxes to confirm that the instructions have indeed been followed.

Digital Taylorism brings about new versions of old control mechanisms, but in reacting to them, workers also borrow from the early 20th century. Reid-Musson, MacEachen, and Bartel (2020, p. 147) compare Uber drivers’ avoidance and mocking of a new carpool service to the “soldiering” steelworkers in 1911, who limited their work effort in response to poor job conditions.

Nevertheless, the control-oriented model is not the only option for a platform company; it is simply the one chosen by many of them. Jarrahi et al. (2020, p. 180) state that Upwork’s knowledge intensive freelancing platform that allows workers to control tasks is an example of how algorithmic management does not necessarily deskill workers. In their study of the food delivery platform, Wolt, Kusk, and Bossen (2022) found that instead of penalizing or reducing wages, the company uses “lenient algorithmic management.” This, combined with providing the possibility of receiving human support in difficult situations, has led to a comparatively positive worker experience. In addition, Wood et al. (2019) note, that in many situations, it is not at all clear what benefit constant surveillance affords companies.

Digital Taylorism is a complex mix of control mechanisms and sociotechnical and economic factors. Whereas sociotechnical factors define the characteristics of digital Taylorism, it is the income dependency on algorithmically managed work that seems to determine its intensity. As low piecemeal wages have been common in algorithmically managed jobs so far, workers are often forced to long hours and to carry out specific, non-flexible, paid, and unpaid tasks to optimize their income. This appears to be the case in both developing and developed countries. The presence of precarious, easily replaceable workers with the status of independent contractor is key to platforms being able to deliver highly standardized services, according to Veen, Barratt, and Goods (2019, p. 401).

One could claim that many of the repetitive, closely controlled jobs envisage further automation. If one day the processes are fully automated, it appears that the companies do not have to greatly change their models when switching from Uber drivers to self-driving taxis, or from food delivery bikers to automatic delivery drones.

#### Asymmetrical information: how algorithmic systems affect organizational power structures

A common theme in the reviewed literature is that in many platform companies, the algorithm “knows” a lot of the worker, but the worker knows little of the algorithm. As Rosenblat and Stark (2016) state, Uber has used this asymmetry of information and power to structure control over its workers. However, information asymmetry is not limited to platform companies. Aloisi and De Stefano (2022) note that since the post-pandemic shift towards remote work practices, any company has at its disposal a plethora of tools from contact-tracing apps to productivity measurement software to gather information on their workers.

Once again, this phenomenon is arguably not an unforeseen circumstance but a new version of old power dynamics. Chan and Humphreys (2018, p. 31) argue that Braverman’s (1974) analysis of managerial control as the abstraction and monopoly of knowledge is relevant to the platform labor of today. Chan and Humphreys (2018) also point out that in the “datafication” of the workplace, knowledge workers, such as journalists, legal experts, and medical professionals, retain more autonomy than blue-collar service workers.

According to Schildt (2017), an aspect of algorithmic management is power shifting from managers to larger groups of experts in analytics, programming, and business. Due to their specific expertise, programmers now have the power to potentially impact millions of workers with their decisions regarding algorithmic management systems. The top management customarily decides the aims of the algorithmic systems, yet the fact that many managers may lack a deep understanding of the code increases the role of programmers. Barati and Ansari (2022) view the lack of technical knowledge as a risk that can lead to overestimating the transparency and objectivity of algorithmic management systems. This can in turn deepen the pre-existing power asymmetries by increasing systematic biases, opacity of decisions and violations of worker privacy (Barati and Ansari 2022).

Workers and employers are not the only stakeholder groups affected by algorithmic technologies. Kinder, Jarrahi, and Sutherland (2019, pp. 212:17–18) describe how the structure of Upwork’s online freelancing platform facilitates information and power asymmetries that favor the company over freelancers and the clients hiring their services. For example, the authors state how Upwork monitors the chat between freelancers and clients, intervening if either party mentions an external platform that could potentially be used to go around Upwork’s intermediary platform.

It is worth stressing that many of the most prominent cases of algorithmic management have taken place in the relatively novel context of the platform economy, where the operational model has been in a state of flux from the start. With the regulations underdeveloped, platform companies have experimented with tweaking their algorithmic systems and operational models—at times in controversial manners. Therefore, scholars have called for updating national and international laws to close these regulatory gaps (Nilsen et al. 2022; Aloisi and De Stefano 2022).

As we have seen, algorithms impact power relationships only partly due to their nature and just as much due to organizational decisions. Bucher, Schou, and Waldkirch (2021, p. 19) emphasize that “algorithms cannot be seen as neutral, technological progress which merely reduces transaction costs between workers and clients”. This position emphasizes the responsibility that companies have in the decision-making process. Accordingly, one can claim that, instead of technology predetermining it, there are several ways in which algorithmic management can be used to manage workers.

### Synthesis: Algorithmic Management Grid – a framework of the way algorithmic systems are used in management

**Fig. 2:**
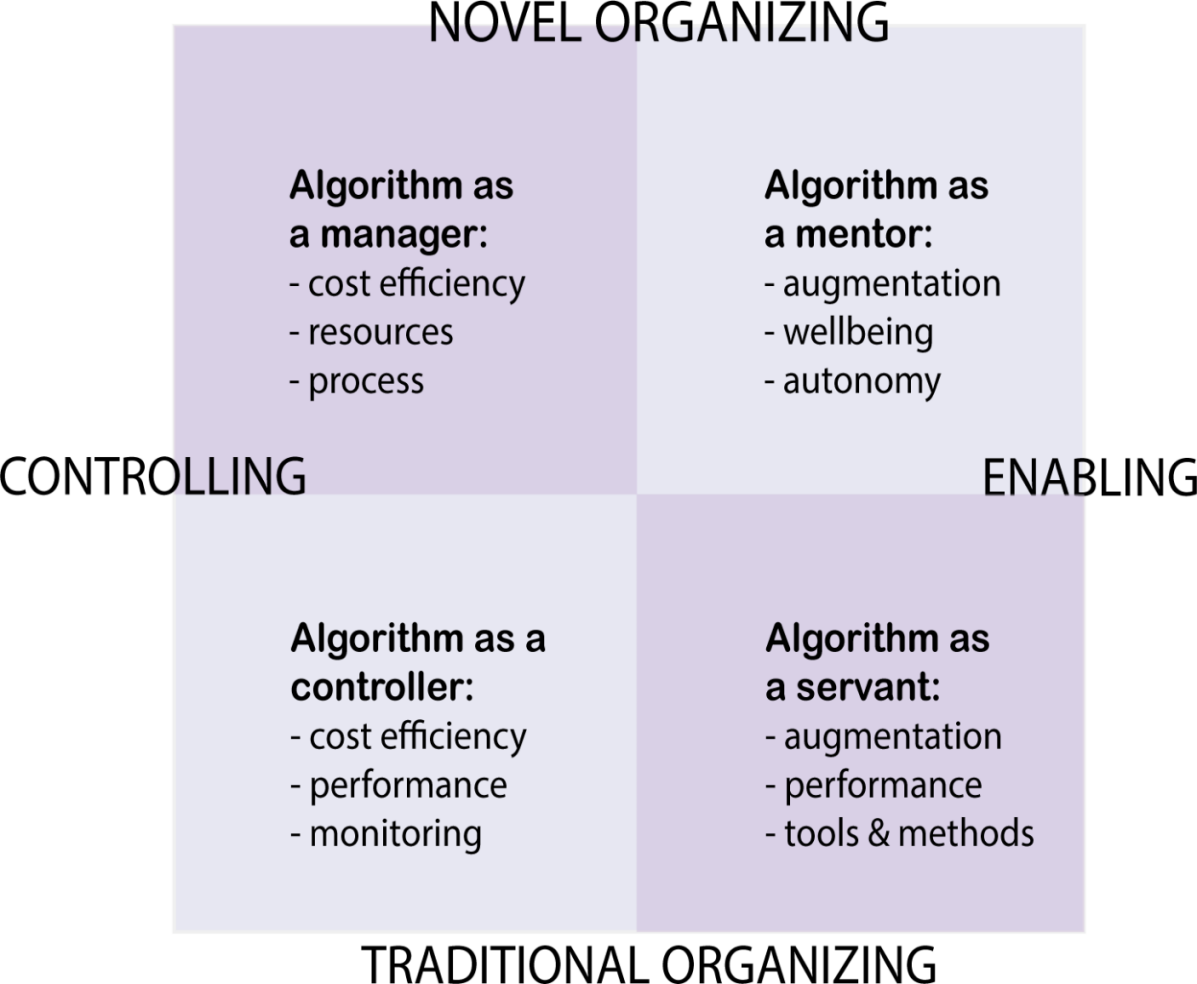
Algorithmic Management Grid: How organizations are using algorithms to manage workers

Based on previous studies’ descriptions of algorithmic management reviewed for this article, we propose a novel framework of Algorithmic Management Grid to categorize the ways algorithms are used to manage workers (Fig. 2). The grid is born from both what exists and what is lacking from the empirical record on the topic. There is an ample amount of case studies describing how algorithmic systems are used in control-oriented ways in platform companies, as well as in some more traditional organizational settings. Yet, mostly lacking from the literature are case studies of more enabling way of using algorithmic systems in management.

Conceptual articles noting the possibility of such arrangements, do however exist. For example, in evaluating algorithms’ role in the future of management, Jarrahi (2019) refers to Zuboff’s (1988) idea of how information technology can either automate work or informate and augment workers. Schildt (2017), in turn, divides algorithmic management systems to optimizing-oriented (with a focus on controlling workers) and open-ended systems (with a focus on providing information for workers). This finding of the theoretical and conceptual imbalance is at the root of our framework.

Thus, the approaches to using algorithmic systems in management are distinguished based on their relation to the two axes of the grid. The horizontal axis is determined by whether an organization uses algorithmic management systems as a tool to closely control and monitor workers, or whether algorithmic systems are provided to workers as a tool to enable and augment them in their work. Schildt’s (2017) division helps to illustrate this distinction. When a system is designed to optimize the behavior of human workers and make decisions for them to follow, it represents the controlling end. If, instead of giving orders, a system provides workers with open-ended information to help make decisions on their own, it is considered an enabling system.

The vertical axis implies whether the algorithmic system is used in a novel or a more traditional model of organizing. The latter implies a model that relies more on human supervisors, whereas in the novel models, most supervisory tasks are automated or in some cases, not carried out at all. While in novel organizing models, such as platform companies, algorithmic systems are more likely in a central, salient role in management, in traditional organizations their role is often limited to carrying out or helping in individual managerial tasks.

#### Controlling – Novel organizing: Algorithm as a manager

Based on the case studies so far, a large chunk of the use of algorithmic management could be classified into the controlling and disruptive quadrant, since many platform companies’ operational model is a relatively novel, disruptive one based on close worker control and monitoring, aimed at optimizing the work process and maximizing performance. Novel methods of organizing are reflected in, for instance, the absence of human managers as well as in the new approaches to work itself—it is less tied to time, location, and organization, representing so-called flexible labor (part-time, short-term, project-based) (see e.g., Edgell et al. 2016). Of the recent examples, food delivery and ride-hailing companies perhaps represent a more controlling end. Some online freelancing companies have more of the enabling features, yet they may remain in the same quadrant. As these novel organizational models rely little on human managers, algorithmic management is perhaps the most distinguishable in this quadrant.

#### Controlling – Traditional organizing: Algorithm as a controller

Both quadrants on the controlling side consist of companies attempting to increase productivity by automating monitoring and controlling functions in ways that may resemble digital Taylorism. The two quadrants have a similar goal but slightly different methods of reaching them. Whereas workers in the novel organizing quadrant are often considered independent contractors, conventional employment relationships and organizational structures are common in the traditional organizing quadrant. In these organizations, the workers interact more with human managers, while the algorithms play a role in monitoring and evaluating their work efficiency and assist human managers in optimization-oriented decision-making. Overall, the traditional methods of organizing are also reflected in the strong connection to time (fixed hours), place (fixed workplace and organization), and rigid labor relationships (fixed contract, longer-term) (see e.g., Autor et al. 2020).

#### Enabling – traditional organizing: Algorithm as a servant

As in the previous quadrant, companies using algorithms as servants are likely to have traditional organizational structures and focus on improving performance. However, the two quadrants have opposing approaches to reaching the target. Using Schildt’s (2017) distinction, the types of algorithmic systems here are more open-ended: instead of a tool that controls workers, it is a tool that workers control. The aim is to equip professionals with algorithmic tools to empower and augment them in their tasks and in making decisions.

For example, IT companies that use project management platforms to facilitate teamwork may be placed in this quadrant. Nevertheless, workers are supervised by human managers, and as such, algorithms’ role is not as pronounced in this quadrant. Workers can face challenges in integrating algorithms into their existing work practices, even while being supportive of their potential (Wolf and Blomberg 2019). This quadrant may include programmers who develop systems that monitor the workers in the controlling quadrants.

#### Enabling – novel organizing: Algorithm as a mentor

Organizations that use algorithms as mentors also attempt to empower and augment workers. However, they do so with novel organizational models, often without a traditional top-down hierarchy, to enable self-directed teams. Platform cooperatives, cooperatively owned companies that utilize a digital platform (see e.g., Schneider 2018) represent an example: cooperative companies that have worker-owners deciding for themselves how to use the algorithms in organizing their work.

It is crucial to note, that by mentor, we do not necessarily imply that the algorithmic system is presented as a virtual, anthropomorphic character. We merely imply that the system provides open-ended information and guidance for workers to base their decisions on — in opposition to a controlling system that makes the decisions for workers to algorithmically execute.

Among the literature on the topic, there are very few cases of using an algorithm as a mentor. The lack of enabling cases in the literature may be explained by the fact that when organizations use digital technology in an empowering manner, it appears to be rarely called algorithmic management. Indeed, there may be numerous organizations around the world using enabling algorithms—perhaps not as technically sophisticated as some of the more controlling ones, but often more effective in achieving their purpose.

The Algorithmic Management Grid aims to show how different the approaches to using algorithms in management may be. However, it is important to note the framework’s limitations. The grid is a simplification; in most cases of algorithmic management, both ends of the grid are at play simultaneously. An algorithm may enable in some areas (e.g., offering flexible working hours) while being simultaneously strictly controlling in others (leaving no flexibility on how to carry out the work). It is often more complicated than the mere sum of the enabling features minus the controlling ones. Even within the same company, two different workers with similar tasks may label their experiences in different quadrants. Furthermore, we do not claim that these two axes are the only ones that could be used to classify algorithmic management. Additionally, how one experiences working under algorithmic management is arguably only partly determined by the way algorithms are used, as other aspects of the work arrangement also appear crucial. These associated aspects, include the level of dependency of platform income and outside factors, such as the price of gasoline for transportation platform workers.

## Discussion & conclusion

### Discussion and suggestions for future research

In this chapter, we discuss the implications of the findings of our systematic literature review and of our theoretical framework, while providing suggestions for future research.

#### Algorithmic management in traditional organizational contexts

The Algorithmic Management Grid was created both by what exists and does not exist among the literature. There exist rich descriptions of working on novel labor platforms under control-oriented algorithmic management systems. Continuing to observe and shape the organizing of platform labour remains important, but more research is needed of the use of algorithmic management systems in more traditional organizational settings (as also pointed by Jarrahi et al. 2021).

Unlike in platform companies, where algorithmic systems are central, in traditional organizational settings the role of algorithms in management is more varied and often limited to helping in individual managerial tasks. Streamlining human resource management processes, such as payroll calculation or recruitment is a common example.

Still, in the aftermath of the remote work boom initiated by the coronavirus pandemic, the influence of algorithmic management systems also appears to be rapidly increasing in traditional organizations. According to news reports (e.g., Marks 2021), a growing number of companies have chosen to use remote monitoring technologies, from in-house cameras to productivity and activity tracking software. This, naturally, opens a host of research questions, from the impact on worker welfare to evaluating whether remote monitoring provides productivity or other benefits.

#### Enabling uses of algorithmic systems in organizing

Also largely missing from the algorithmic management literature – and what we aimed to highlight with our framework – is technology’s potential to enable novel, autonomous forms of organizing. It appears that with the rise of platform labor and remote work arrangements, the future of work will look different than during the last century. The threats and perils along this trajectory are aptly documented (e.g., Huws 2016; Fleming 2017; Faraj et al. 2018), yet it remains less clear what the desired future of organizing looks like. For example, the way the Dutch nursing company Buurtzorg utilizes technology in supporting its self-directed teams of 12 nurses (see e.g., Nandram and Koster 2014) provides an interesting contraposition to the control-oriented platform companies, which is lacking in the algorithmic management literature. How this gap may be bridged empirically and conceptually is a crucial avenue for future studies.

In addition, as the enabling uses of algorithmic systems may not be called algorithmic management, it is likely that there exist studies that use different terms to describe cases where technology is used in support of self-directed teams and shared leadership, for example. Therefore, future studies should focus on bringing existing contributions from such discussions into the debate on algorithmic management, in addition to studying novel empirical contexts.

#### Extending algorithmic management research to new contexts and targets

There are various contexts for more algorithmic management research. These contexts include previously understudied countries and types of case organizations around the world.

Even though there are studies on the topic from other contexts as well (e.g., Bai et al. 2020; Basukie et al. 2020; Anwar et al. 2021), much of the research has taken place in Western contexts and is written from a Western point of view. Yet, as algorithmic management practices on crowdwork platforms create a global on-demand workforce (Altenried 2020), more research from local viewpoints around the world are needed, for example on the experiences of working for European or North American companies from the Global South.

Another interesting context largely missing from the discussion on algorithmic management is platform cooperatives (see e.g., Schneider 2018). Studying and comparing how workers perceive algorithmic management systems in platform cooperatives could provide valuable insights on the phenomenon.

Previous studies have justly focused on workers’ perceptions of algorithmic management, but the scrutiny should be extended to managers, software developers and other stakeholders. Even though software developers’ role has been identified as increasingly influential in shaping algorithmic management (Schildt 2017; Jarrahi et al. 2021), few studies have examined their perspective on the topic. As such, it is critical to conduct more studies where software developers’ perceptions of the process of developing algorithmic systems is interpreted.

#### Algorithmic management in relation to traditional management theories

Evaluating algorithmic management through the framing of digital Taylorism has allowed to situate the practices along the progression of organization theories. Algorithmic Management Grid provides opportunities for approaching the topic through the lens of traditional management theories beyond Taylorism. For example, future studies can examine and compare the sensemaking processes (e.g., Weick et al. 2005) of workers, software developers and managers in companies located at different quadrants of the grid. Also, future studies should address the potential short-term and long-term implications on efficiency, welfare, and innovativeness of organizations by different approaches to algorithmic management.

Additionally, future research should further explore the role of technology in relation to the general approach to organizing. For example, what is more important in reaching desired organizational outcomes, utilizing the most advanced algorithms or the general approach to organizing? We believe that the decision on whether algorithmic systems are used to control or enable workers is central on the matter. In addition, the role of culture remains greatly understudied, and future studies should address how organizational culture influences or is influenced by different ways of utilizing algorithmic management.

#### Factors impacting workers’ perceptions of algorithmic management

The articles reviewed in our article show a variety of organizational use cases and workers’ experiences under algorithmic management. These experiences seem to be partly explained by the algorithmic management system itself, and partly by other factors surrounding it, such as, income dependency, employment status and the location of worker and the company. More research is needed to determine the impact each factor has on working under algorithmic management. A variety of approaches and methods seem suitable. For example, a qualitative approach can identify the factors impacting work under algorithmic management and a quantitative approach may be used to investigate the factors’ relative importance.

Although not the whole picture, the ongoing debate on the employment status of platform workers seems to be a crucial piece in the puzzle of algorithmic management and novel models of organizing. Traditionally, an entrepreneur has been justified to earn a profit as a reward for assuming the financial risk involved. An employee, on the contrary, trades the chance of profit for the security of making a steady living with less financial risk. In the worst case, however, platform workers under algorithmic management receive the short end of both sticks: they have neither the possibility of making a profit nor the security of making a steady living.

Income dependency also appears to be a key determinant of the experience of working under algorithmic management. One could claim that even if all other issues, from employment status to coercive control, were solved, it would be for naught if the compensation from algorithmically managed work was not enough to pay the bills. However, making a living is still only the baseline motivation, and gig workers naturally have a variety of other motivational goals, as evidenced by Jabagi et al. (2019).

#### Algorithmic management and the future of work

In the past years, there has been speculation about digitalization and artificial intelligence wiping out workers across a variety of fields (e.g., Frey and Osborne 2017). However, so far, one could claim that instead of replacing workers with robots, workers have often been forced to work as if they were robots, and the tasks being replaced are largely the ones of managers of these “human robots”. Indeed, in his examination of algorithmic management among the historical development between capital, labor, and investment in automation, Schaupp (2022) argues that algorithmic management has been viewed as a cheaper alternative to automation. As fully automating production processes demands heavier investment, companies have instead chosen algorithmic management practices to gain more control over the workforce, that remains more flexible than robots.

Nevertheless, in mechanical tasks, there is arguably nothing inherently wrong with using algorithmic management in a more controlling manner (see Lee 2018). In fact, an enabling manner could needlessly complicate tasks, such as connecting the nearest driver to pick up the passenger and choosing the fastest route to their destination. Based on the literature, however, the problems with working under algorithmic management seem to arise when workers are treated as if they were robots, not acknowledging their human needs and limitations.

It should also be noted that in most companies, only some of the tasks of the organization seem to be performed by algorithms. The rest are done by either human managers, workers themselves, or not done at all, as often seems the case with providing organizational support, for example.

As managerial tasks are reimagined through algorithmic systems, platform companies’ bare-bones organizational models allow both theorists and practitioners to re-examine fundamental questions, such as whether human supervisors are needed or can responsibility and leadership be shared among workers. Algorithmic technologies further emphasize the importance of the rules and regulations with which organizations operate. Therefore, the decision on how algorithmic systems are used in management is essential.

Although rapidly developing, the topic of algorithmic management has not reached a level of full maturity. Will algorithmic management practices and its theories experience similar stages or paradigms as the discipline of human management did, going from efficiency maximizing Taylorism to more human-centered approaches? In any case, by learning from the missteps and accomplishments of the early versions of algorithmic management, theorists and practitioners may have a chance to ease some of the growing pains of the process.

## Conclusion

Despite being a rapidly expanding research area, little summarizing literature exists on the way algorithmic systems are used in management. This systematic literature review complements the insights provided by previous reviews on the topic (Kellogg et al. 2020; Heinrich et al. 2022), by (1) providing a descriptive overview of algorithmic management literature, (2) by identifying and analyzing the key concepts among the debate on algorithmic management, and (3) by creating a theoretical framework, Algorithmic Management Grid, that depicts the ways organizations are using algorithmic systems in management.

We identified the following key debates among the topic: First, algorithmic management practices simultaneously restrain and enable workers’ autonomy (e.g., Lee et al. 2015; Möhlmann and Zalmanson 2017) yet the need to make ends meet seems to force workers to long days and specific work practices (e.g., Wood et al. 2019; Schor et al. 2020). Second, algorithmic management systems create a digital version of Taylorism, with both new and old characteristics (e.g., Rosenblat and Stark 2016; Schildt 2017; Altenried 2020). Third, algorithmic management practices and its built-in information asymmetry create new and changes existing power structures (e.g., Rosenblat and Stark 2016; Gandini 2019; Jarrahi et al. 2020).

While previous studies have advanced the debate on the topic with various conceptual contributions, models depicting different types of algorithmic management systems have been lacking. Our framework of Algorithmic Management Grid shows how algorithmic systems may be used with a focus on either controlling or enabling workers in both platform companies and more traditional organizations.

Ultimately, the way algorithmic management is used appears to be determined by trust—whether workers are trusted to perform well autonomously or whether workers need to be controlled and monitored to make sure they are doing what they are supposed to. Based on the reviewed studies, it seems that algorithmic management has so far been mostly used in a controlling, exploitative manner. We claim, however, that this need not be the case, as there exist more enabling and less exploitative ways of using algorithmic systems in the organization of work. The Algorithmic Management Grid emphasizes that the way companies use algorithmic management is not predetermined by technology but decided by organizational actors.

Algorithmic management tools may be used as a support system that provides flexibility and efficiency by minimizing organizational hierarchy. However, in the worst case, algorithmic tools are used to bring the oppressive principles of scientific management to the extreme, resulting in Taylorism on steroids.

**Statements and Declarations**.

## Data Availability

The data that support the findings of this study are available from the corresponding author upon request.

## References

[CR1] Agarwal N, Steinmetz R (2019) Sharing economy: a systematic literature review. Int J Innov Technol Manage 16. 10.1142/S0219877019300027

[CR2] Aloisi A, De Stefano V (2022). Essential jobs, remote work and digital surveillance: addressing the COVID-19 pandemic panopticon. Int Labour Rev.

[CR3] Altenried M (2019). On the last mile: logistical urbanism and the transformation of labour. Work Organisation. Labour & Globalisation.

[CR4] Altenried M (2020). The platform as factory: Crowdwork and the hidden labour behind artificial intelligence. Capital & Class.

[CR5] Anwar IA, Pal J, Hui J (2021). Watched, but moving: platformization of Beauty Work and its gendered mechanisms of control. Proc ACM Hum Comput Interact.

[CR6] Autor DH, Mindell DA, Reynolds EB (2020). The work of the future: building better jobs in an age of intelligent machines.

[CR7] Bai B, Dai H, Zhang D et al (2020) The impacts of algorithmic work assignment on Fairness perceptions and Productivity: evidence from Field experiments. 10.2139/ssrn.3550887. SSRN Electronic Journal

[CR8] Barati M, Ansari B (2022). Effects of algorithmic control on power asymmetry and inequality within organizations. J Manage Control.

[CR9] Basukie J, Wang Y, Li S (2020). Big data governance and algorithmic management in sharing economy platforms: a case of ridesharing in emerging markets. Technol Forecast Soc Change.

[CR10] Braesemann F, Stephany F, Teutloff O et al (2022) The global polarisation of remote work. PLoS ONE 17. 10.1371/JOURNAL.PONE.0274630 10.1371/journal.pone.0274630 PMC9584402 36264859

[CR11] Braverman H (1974). Labor and Monopoly Capital: the degradation of work in the Twentieth Century.

[CR12] Bucher EL, Schou PK, Waldkirch M (2021) Pacifying the algorithm – Anticipatory compliance in the face of algorithmic management in the gig economy. Organization. 10.1177/1350508420961531

[CR13] Cameron L, Lamers L, Leicht-Deobald U et al (2023) Algorithmic Management: its implications for Information Systems Research. Communications of the Association for Information Systems 52

[CR14] Chan NK, Humphreys L (2018). Mediatization of social space and the case of uber drivers. Media Commun.

[CR15] Cheng M (2016). Sharing economy: a review and agenda for future research. Int J Hosp Manag.

[CR16] Cheng MM, Hackett RD (2021). A critical review of algorithms in HRM: definition, theory, and practice. Hum Resource Manage Rev.

[CR17] Dekker R, Koot P, Birbil SI et al (2022) Co-designing algorithms for governance: Ensuring responsible and accountable algorithmic management of refugee camp supplies. Big Data Soc 1–15. 10.1177/20539517221087855

[CR18] Dillahunt TR, Wang X, Wheeler E (2017). The sharing economy in Computing: a systematic literature review. Proc ACM Hum Comput Interact.

[CR19] Duggan J, Sherman U, Carbery R, McDonnell A (2020). Algorithmic management and app-work in the gig economy: a research agenda for employment relations and HRM. Hum Resource Manage J.

[CR20] Edgell S, Gottfried H, Granter E (2016). The SAGE handbook of the sociology of work and employment.

[CR21] Faraj S, Pachidi S, Sayegh K (2018). Working and organizing in the age of the learning algorithm. Inf Organ.

[CR22] Fayol H (1917) General and Industrial Management. Dunod et E. Pinat

[CR23] Fisch C, Block J (2018). Six tips for your (systematic) literature review in business and management research. Manage Rev Q.

[CR24] Fleming P (2017). The human capital hoax: work, debt and insecurity in the era of uberization. Organ Stud.

[CR25] Frey CB, Osborne MA (2017). The future of employment: how susceptible are jobs to computerisation?. Technol Forecast Soc Change.

[CR26] Gagné M, Parent-Rocheleau X, Bujold A (2022). How algorithmic management influences worker motivation: a self-determination theory perspective. Can Psychol.

[CR27] Galière S (2020). When food-delivery platform workers consent to algorithmic management: a foucauldian perspective. New Technol Work Employ.

[CR28] Gandini A (2019). Labour process theory and the gig economy. Hum Relat.

[CR29] Giermindl LM, Strich F, Christ O et al (2021) The dark sides of people analytics: reviewing the perils for organisations and employees. https://doi.org/101080/0960085X20211927213

[CR30] Glikson E, Woolley AW (2020). Human trust in artificial intelligence: review of empirical research. Acad Manag Ann.

[CR31] Gómez-Zará D, Das A, Pawlow B, Contractor N (2022). In search of diverse and connected teams: a computational approach to assemble diverse teams based on members’ social networks. PLoS ONE.

[CR32] Görög G (2018) The definitions of sharing economy: a systematic literature review. Management 175–189. 10.26493/1854-4231.13.175-189

[CR33] Grint K (2011) A history of leadership. In: Bryman A, Collinson D, Grint K et al (eds) The SAGE handbook of leadership, 14th edn. SAGE, pp 3–14

[CR34] Heinrich K, Minh Anh V, Vysochyna A (2022) Algorithms as a Manager: A Critical Literature Review of Algorithm Management. ICIS 2022 Proceedings

[CR35] Huws U (2016). Logged labour: a new paradigm of work organisation? Work Organisation. Labour and Globalisation.

[CR36] Jabagi N, Croteau AM, Audebrand LK, Marsan J (2019). Gig-workers’ motivation: thinking beyond carrots and sticks. J Managerial Psychol.

[CR37] Jarrahi MH (2019). In the age of the smart artificial intelligence: AI’s dual capacities for automating and informating work. Bus Inform Rev.

[CR39] Jarrahi MH, Sutherland W (2019) Algorithmic Management and Algorithmic Competencies: understanding and appropriating algorithms in gig work. Lecture notes in Computer Science (including subseries lecture notes in Artificial Intelligence and Lecture Notes in Bioinformatics). Springer Verlag, pp 578–589

[CR40] Jarrahi MH, Sutherland W, Nelson SB, Sawyer S (2020). Platformic Management, Boundary Resources for Gig Work, and worker autonomy. Comput Support Coop Work.

[CR38] Jarrahi MH, Newlands G, Lee MK et al (2021) Algorithmic Management in a work context. Big Data Soc

[CR41] Kaine S, Josserand E (2019). The organisation and experience of work in the gig economy. J Industrial Relations.

[CR42] Kässi O, Lehdonvirta V, Stephany F (2021) How many online workers are there in the World? A Data-Driven Assessment. 10.2139/SSRN.3810843. SSRN Electronic Journal 10.12688/openreseurope.13639.4 PMC10445891 37645214

[CR43] Keding C (2021). Understanding the interplay of artificial intelligence and strategic management: four decades of research in review. Manage Rev Q.

[CR44] Kellogg KC, Valentine MA, Christin A (2020). Algorithms at work: the new contested terrain of control. Acad Manag Ann.

[CR45] Kinder E, Jarrahi MH, Sutherland W (2019). Gig platforms, tensions, alliances and ecosystems: an actor-network perspective. Proc ACM Hum Comput Interact.

[CR46] Kusk K, Bossen C (2022) Working with Wolt. Proc ACM Hum Comput Interact 6. 10.1145/3492823

[CR47] Lee MK (2018). Understanding perception of algorithmic decisions: Fairness, trust, and emotion in response to algorithmic management. Big Data Soc.

[CR48] Lee MK, Kusbit D, Metsky E, Dabbish L (2015) Working with Machines. In: Proceedings of the 33rd Annual ACM Conference on Human Factors in Computing Systems. 1603–1612. ACM Press, New York

[CR49] Ma NF, Hanrahan B (2019). Part-time ride-sharing: recognizing the context in which drivers ride-share and its impact on platform use. Proc ACM Hum Comput Interact.

[CR50] Marks G (2021) Want to keep tabs on your working-from-home staff? Resist the urge. The Guardian

[CR51] Meijerink J, Bondarouk T (2021) The duality of algorithmic management: toward a research agenda on HRM algorithms, autonomy and value creation. Hum Resource Manage Rev 100876. 10.1016/J.HRMR.2021.100876

[CR52] Mengay A (2020). Digitalization of work and heteronomy. Capital & Class.

[CR53] Möhlmann M, Zalmanson L (2017) Hands on the Wheel: Navigating Algorithmic Management and Drivers. In: Proceedings of the International Conference on Information Systems (ICIS 2017), December 10–13, Seoul, South Korea

[CR54] Muralidhar SH, Bossen C, O’Neill J (2022). Between a Rock and a hard place: negotiating dependencies and precarity in the On-Demand economy. Comput Support Coop Work.

[CR55] Nandram S, Koster N (2014). Organizational innovation and integrated care: Lessons from Buurtzorg. J Integr Care.

[CR56] Nilsen M, Kongsvik T, Antonsen S (2022). Taming Proteus: Challenges for risk regulation of powerful Digital Labor Platforms. Int J Environ Res Public Health 2022.

[CR57] Ovetz R (2020) The Algorithmic University: On-Line Education, Learning Management Systems, and the Struggle over Academic Labor. Crit Sociol (Eugene). 10.1177/0896920520948931

[CR58] Parent-Rocheleau X, Parker SK (2022). Algorithms as work designers: how algorithmic management influences the design of jobs. Hum Resource Manage Rev.

[CR59] Perez F, Conway N, Roques O (2022) The autonomy tussle: AI technology and employee job crafting responses. Relations industrielles 77. 10.7202/1094209AR

[CR60] Pignot E (2021) Who is pulling the strings in the platform economy? Accounting for the dark and unexpected sides of algorithmic control: https://doi.org/101177/1350508420974523

[CR61] Rani U, Furrer M (2021) Digital labour platforms and new forms of flexible work in developing countries: Algorithmic management of work and workers. Compet Change. 10.1177/1024529420905187

[CR62] Reid-Musson E, MacEachen E, Bartel E (2020). Don’t take a poo!’’: worker misbehaviour in on-demand ride-hail carpooling. New Technol Work Employ.

[CR63] Rosenblat A, Stark L (2016). Algorithmic labor and information asymmetries: a case study of Uber’s drivers. Int J Commun.

[CR64] Ruiner C, Klumpp M (2022). Autonomy and new modes of control in digital work contexts-a mixed-methods study of driving professions in food logistics. Empl Relations.

[CR65] Sahlin J, Angelis J (2019) Performance management systems: reviewing the rise of dynamics and digitalization. Cogent Bus Manage 6. 10.1080/23311975.2019.1642293. :

[CR66] Schaupp S (2022). COVID-19, economic crises and digitalisation: how algorithmic management became an alternative to automation. New Technol Work Employ.

[CR67] Schildt H (2017). Big data and organizational design - the brave new world of algorithmic management and computer augmented transparency. Innovation.

[CR68] Schneider N (2018). An internet of ownership: democratic design for the online economy. Sociol Rev.

[CR69] Schor JB, Attwood-Charles W, Cansoy M (2020). Dependence and precarity in the platform economy. Theory Soc.

[CR70] Snyder H (2019). Literature review as a research methodology: an overview and guidelines. J Bus Res.

[CR71] Stark D, Pais I (2020). Algorithmic Management in the platform economy. Sociologica.

[CR72] Terry E, Marks A, Dakessian A, Christopoulos D (2021) Emotional Labour and the Autonomy of Dependent Self-Employed Workers: The Limitations of Digital Managerial Control in the Home Credit Sector. https://doi.org/101177/0950017020979504 36:665–682

[CR73] Tranfield D, Denyer D, Smart P (2003). Towards a methodology for developing evidence-informed management knowledge by means of systematic review. Br J Manag.

[CR74] Veen A, Barratt T, Goods C (2019) Platform-capital’s ‘App-etite’ for control: a labour process analysis of Food-Delivery Work in Australia: https://doi.org/101177/0950017019836911 34. 388–406. 10.1177/0950017019836911

[CR75] Webster J, Watson RT (2002). Analyzing the past to prepare for the future: writing a literature review. MIS Q.

[CR76] Weick KE, Sutcliffe KM, Obstfeld D (2005) Organizing and the Process of Sensemaking. https://doi.org/101287/orsc10500133. 16:409–421.

[CR77] Weili LIU, Khan H (2020) A literature review on the definition of sharing economy. Global Econ J 20. 10.1142/S219456592030001X

[CR78] Wolf CT, Blomberg JL (2019). Evaluating the promise of human-algorithm collaborations in everyday work practices. Proc ACM Hum Comput Interact.

[CR79] Wood AJ, Graham M, Lehdonvirta V, Hjorth I (2019). Good gig, bad gig: autonomy and Algorithmic Control in the global gig economy. Work Employ Soc.

[CR80] Zhu X, Liu K (2020). A systematic review and future directions of the sharing economy: business models, operational insights and environment-based utilities. J Clean Prod.

[CR82] Zuboff S (1988). In the age of the smart machine: the future of work and power.

[CR81] Zuboff S (2019). The age of surveillance capitalism: the fight for a human future at the new frontier of power.

